# Treg and intestinal myofibroblasts-derived Amphiregulin induced by TGF-β mediates intestinal fibrosis in Crohn’s disease

**DOI:** 10.1186/s12967-025-06413-6

**Published:** 2025-04-17

**Authors:** Lu Wang, Shu Wang, Junjie Lin, Jiajia Li, Mingyuan Wang, Jiang Yu, Junjian Sun, Nana Tang, Chunhua Jiao, Jingjing Ma, Xiaojing Zhao, Hongjie Zhang

**Affiliations:** Department of Gastroenterology, First Affiliated Hospital with Nanjing Medical University, Nanjing, 210029 China

**Keywords:** Crohn’s disease, Intestinal fibrosis, Tregs, Fibroblast, Amphiregulin

## Abstract

**Background:**

Intestinal fibrosis is a serious complication of Crohn’s disease (CD), often resulting from chronic inflammation. However, the precise mechanisms through which inflammation induces intestinal fibrosis remain inadequately elucidated.

**Methods:**

A comprehensive single-cell atlas of full-thickness CD, provided by Dr. Florian Rieder, was subjected to reanalysis. Our study used a DSS-induced chronic colitis model in both wild-type (WT) and *Areg*^*−/−*^ mice. Additionally, a CD45RB^hi^ CD4^+^ T cell adoptive transfer model involving WT and *Areg*^*−/−*^ Treg cells (Tregs) was used. The expressions of AREG in CD with or without intestinal fibrosis, Tregs and human intestinal myofibroblasts (MFs) were determined. The effect of AREG on proliferation/migration/activation in human intestinal MFs was determined.

**Results:**

Several types of cells were differentially expressed between stricture and non-stricture CD. Among T cells, Tregs accounted for a larger proportion and were significantly increased in stenotic tissues of stricture CD. Although DSS-induced colitis was more severe in *Areg*^*−/−*^ mice, which developed less severe intestinal fibrosis compared with WT mice. The transfer of *Areg*^*−/−*^ Tregs resulted in less severe fibrosis in *Rag*^*−/−*^ mice than WT Tregs. Moreover, TGF-β stimulated AREG expression in Tregs and human intestinal MFs via activation of Smad3.

**Conclusion:**

These findings demonstrated that AREG derived from Tregs and human intestinal MFs, induced by TGF-β, amplifies intestinal fibrotic reactions in experimental colitis as well as in human CD patients. Thus, the TGF-β-Smad3-AREG pathway could be a potential therapeutic target for treating fibrosis in CD.

**Supplementary Information:**

The online version contains supplementary material available at 10.1186/s12967-025-06413-6.

## Introduction

Crohn’s disease (CD) is characterized by chronic, persistent intestinal inflammation resulting in transmural tissue damage [1]. With an incidence rate ranging from 3 to 20 cases per 100,000 individuals, CD represents a significant clinical challenge [2]. Intestinal fibrosis, a common and serious complication of CD, results from abnormal wound healing and is characterized by an increased number of mesenchymal cells and excessive extracellular matrix (ECM) production [3, 4]. It is estimated that > 50% of patients with CD develop clinically apparent fibrostenosing lesions in their lifetime [4]. Conventional anti-inflammatory therapies offer only moderate reduction or deceleration of stenotic disease progression. Currently, no effective anti-fibrotic or anti-stenotic treatments exist, with surgical or endoscopic interventions remaining the primary options for managing CD with fibrotic strictures [3].

The association between inflammation and fibrosis has been discussed for decades, with growing evidence underscoring the role of intestinal inflammation in inducing intestinal fibrosis in CD patients. Intestinal inflammation plays a pivotal role in both triggering and promoting intestinal fibrosis, primarily by activating myofibroblasts (MFs) and subsequently driving the synthesis of ECM [5]. This activation process, analogous to immune cell activation, involves significant changes in cellular function and marker expression. Once activated, myofibroblasts (vimentin^+^, α-SMA^+^, desmin^−^) synthesize higher level ECM and exhibit a higher contractile ability than fibroblasts [6]. Traditionally, fibrosis is perceived as a slow and irreversible process initiated by inflammation. Under normal physiological conditions, acute inflammation is usually followed by tissue repair and wound healing, which is considered as a self-limited process of overcoming tissue damage and loss. Conversely, in pathological states, fibrosis arises from repeated or sustained tissue damage, leading to the failure of structural and functional tissue repair. The interaction between inflammation and MFs has been demonstrated to play an essential role in the development of intestinal fibrosis, especially in CD patients. The chronic intestinal inflammation involves both innate and adaptive immune responses, which are essential in driving the shift from normal tissue repair to fibrotic outcomes. These immune responses also contribute to the activation of MFs generated by ECM, thereby promoting the development and progression of intestinal fibrosis.

T cells are implicated in the pathogenesis of CD, however, their involvement in intestinal fibrogenesis is not as well understood. In recent years, advancements in single-cell analytics have expanded the opportunities for research in CD, facilitating the acquisition of a more comprehensive view of the cellular landscape [7]. However, prior single-cell studies have primarily focused on intestinal inflammation in CD. Recently, Dr. Rieder conducted the first single-cell RNA sequencing (scRNA-seq) on stenotic intestinal tissues in CD, offering a more profound insight into the immune cellular specificity within the intestinal tissues of stricturing CD [8]. Based on the single-cell data provided by Dr. Rieder, we identified differential expression of several cell types between stricturing and non-stricturing CD. Notably, regulatory T cells (Treg cells, Tregs) constituted a larger proportion among the T cell populations, with their numbers being higher in stricturing CD compared to non-stricturing CD. Indeed, multiple studies have indicated that Tregs exert an inhibitory effect on tissue fibrosis [9, 10]. For example, in pulmonary fibrosis, Tregs are associated with improvement in idiopathic pulmonary fibrosis (IPF) [9]. However, Tregs are also an important source of TGF-β, some studies suggest that TGF-β-producing Tregs are involved in inducing rather than suppressing fibrosis [11]. Our previous study found that Th17-cell derived amphiregulin (AREG) is involved in intestinal fibrosis, and Tregs were found to highly express AREG [12]. Current studies investigating the role of Tregs in intestinal fibrosis mainly focus on the TGF-β secreted by Tregs. In fact, few studies have examined the mechanisms by which Tregs mediate intestinal fibrosis [13]. The role and mechanism of AREG derived from Tregs in intestinal fibrosis have not yet been reported.

In this study, we explored the mechanisms involving Tregs and human intestinal MFs in the development of intestinal fibrosis. We noted a greater number of Tregs in CD patients with strictures than in those without strictures. TGF-β promoted AREG secretion in Tregs and MFs through activation of Smad3, a key factor in the induction of intestinal fibrosis [14]. Notably, when Tregs deficient in Areg transferred into *Rag*^*−/−*^ mice induced less severe fibrosis.

## Materials and methods

The data, methodologies, and study materials will be available to other researchers to reproduce our findings or replicate the procedures. Further detailed methods are provided in the Supplemental Material.

### Mice

Specific pathogen-free (SPF) C57BL/6 wild-type (WT) mice (Catalog No.N000013, GemPharmatech) and *Rag*^−/−^ mice (Catalog No.T004753, GemPharmatech) were purchased from GemPharmatech, Inc. and housed in the animal facilities at Nanjing Medical University. *Areg*^*−/−*^ mice and their WT sibling littermates, generated from *Areg*^*+/−*^ x *Areg*^*+/−*^ crosses, were developed by the Shanghai Model Organisms Center, Inc. and housed at the animal facilities of Nanjing Medical University. The Experimental Animal Care and Ethics Committee with Nanjing Medical University reviewed and approved all animal experiments (IACUC-2312007).

### Chronic colitis model generated using dextran sulfate sodium (DSS)

WT and *Areg*^*−/−*^ mice were administered 1.5% dextran sulfate sodium (DSS) in their drinking water for a week, followed by regular drinking water, and the above process was repeated three times [12]. Mice were euthanized following three cycles of DSS treatment.

### T-cell transfer model

CD45RB^hi^ CD4^+^ T cell adoptive transfer model was used to induce chronic colitis in *Rag*^*−/−*^ mice. Meanwhile, splenic CD4^+^ T cells of *Areg*^*−/−*^ and WT mice were extracted and cultured under Treg-polarization conditions for five days. These polarization conditions, which drive the acquisition of distinct functional phenotypes in response to specific cytokine milieus, enabled the differentiation of naive CD4^+^ T cells into Tregs. On day 5, 1 × 10^^5^ CD45RB^hi^ CD4^+^ T with 1 × 10^^6^ WT or *Areg*^*−/−*^ Tregs were transferred into *Rag*^*−/−*^ mice via tail vein [15]. Six weeks following the cell transfer, the mice were euthanized.

### Human tissues and cells

Samples of human intestinal tissue were obtained from individuals with CD, both with and without fibrotic conditions, by the Surgery Department at the First Affiliated Hospital with Nanjing Medical University. Intestinal fibrosis was determined according to the previous study [16]. Peripheral blood samples were collected from CD patients, including both those with and without intestinal fibrosis. Surgical specimens from CD patients with strictures were collected from both the stenotic and adjacent nonstenotic areas of the ileum. The clinical characteristics for CD patients are detailed in Supplementary Table 1. This study was approved by the Ethics Committee of the First Affiliated Hospital with Nanjing Medical University (2023-SR-852).

### Histopathological analysis

Colonic tissue samples were preserved in 10% formaldehyde. The sections embedded in paraffin were then stained following a standard hematoxylin and eosin (H&E) procedure. These H&E-stained sections were assessed for intestinal inflammation using criteria such as crypt or mucosa destruction, the extent of colon involvement, lymphocyte infiltration, and disruption of goblet cells. Each criterion was assessed using a scale ranging from 0 to 3, with 0 representing absence, 1 representing mild, 2 representing moderate, and 3 representing severe. The total inflammatory score was determined by summing the individual scores. And the analyses were independently assessed by two researchers employing a blinded methodology.

### Fibrosis analysis

To evaluate changes in the ECM and collagen deposition, paraffin-embedded sections were stained with Masson’s Trichrome according to standard protocols, as previously described [17]. Collagen thickness was evaluated in sections stained with immunofluorescence, following a previously outlined protocol [18]. The analyses were independently assessed by two researchers employing a blinded methodology.

### Statistical analysis

GraphPad Prism 8.0 (GraphPad Software, USA) was used for statistical analysis, graphing, and calculations. The data are presented as the mean ± standard error of the mean (SEM). Analyses were conducted using Student’s t-test or one-way analysis of variance (ANOVA), followed by Tukey’s post-hoc test. A *P*-value below 0.05 was regarded as statistically significant.

## Results

### A single-cell atlas of full-thickness CD reveals a higher number of Treg cells in stricturing CD

**Fig. 1:**
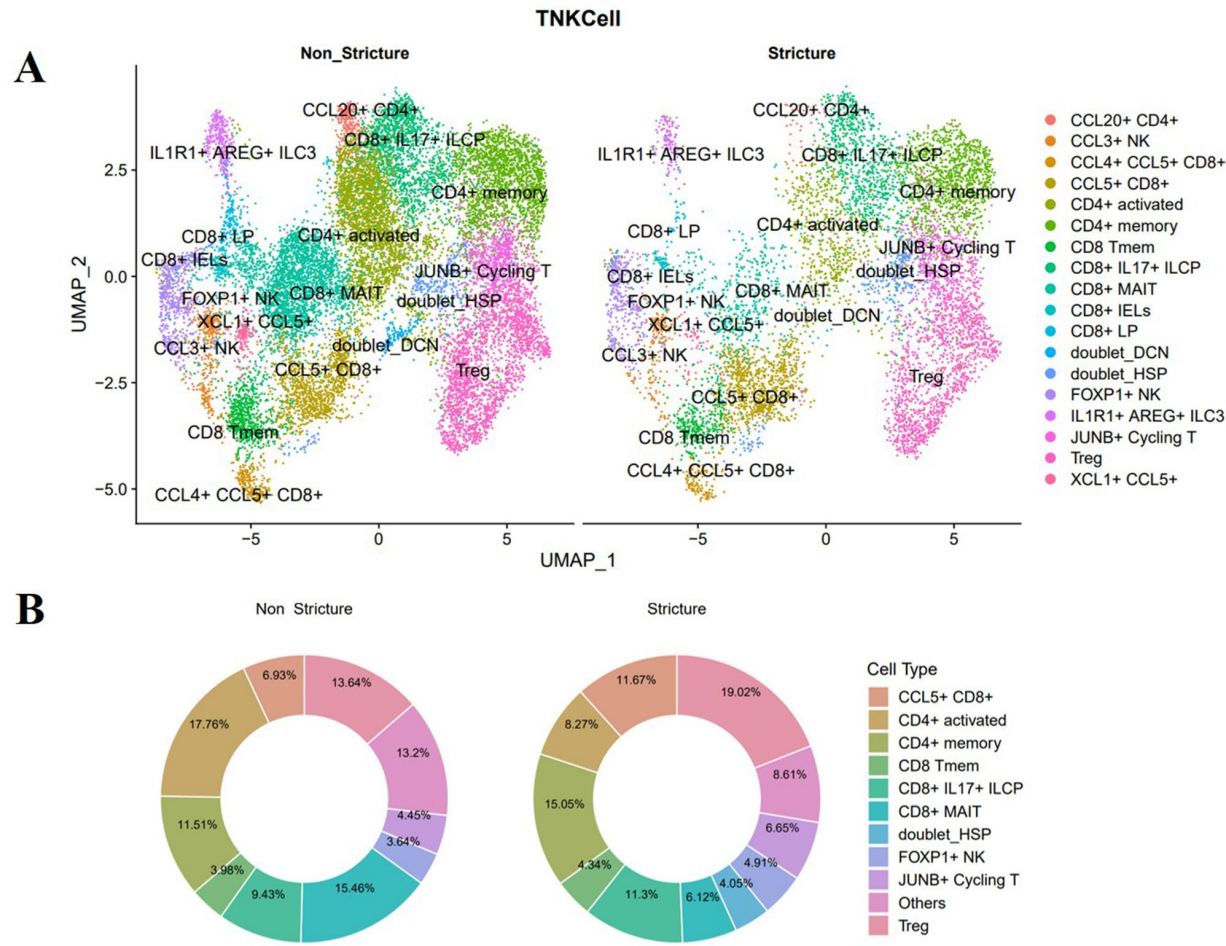
A single-cell atlas of full-thickness CD reveals higher numbers of Treg cells in stricturing Crohn’s disease. We got the scRNAseq data from Dr. Florian Rieder of Lerner Research Institute, Cleveland Clinic. Freshly resected full-thickness intestinal specimens, including strictured (CDs), nonstrictured (CDi), and noninvolved (CDni) tissues, were collected from CD patients. Both noninvolved and inflamed nonstrictured were classified as nonstrictured, and finally divided into two groups: stricture VS nonstricture. (**A**) Several types of cells were differentially expressed between stricture CD with nonstricture CD. (**B**) Different T cell ratios in stricture and nonstricture CD

T cells have been implicated in the pathogenesis of intestinal inflammation in CD. To explore the role of T cells in inducing intestinal fibrosis, we got the scRNAseq data from Dr. Florian Rieder at the Lerner Research Institute, Cleveland Clinic. Freshly resected full-thickness intestinal specimens from CD (strictured (CDs), nonstrictured (CDi), and noninvolved (CDni) tissues). The transcriptional atlas was generated from 13 ileal CD full-thickness. Due to the total cell count being fewer than 100 in two CD patients (CD2577 and CD2586), these were excluded from the final analysis. Finally, we analyzed the transcriptional atlas from 11 ileal CD full-thickness, with an extremely significant difference defined as FDR < 0.01. Non-involved and inflamed non-strictured tissues were categorized as non-strictured, resulting in two groups: stricture vs. non-stricture. Several types of cells were differentially expressed between stricture CD with non-stricture CD (Fig. 1A). Among T cells, Tregs constituted a larger proportion and were significantly increased in the stenotic tissues of strictured CD (Fig. 1B), suggesting that Tregs may play a crucial role in intestinal fibrosis of CD.

### Treg cells produce high levels of AREG

**Fig. 2:**
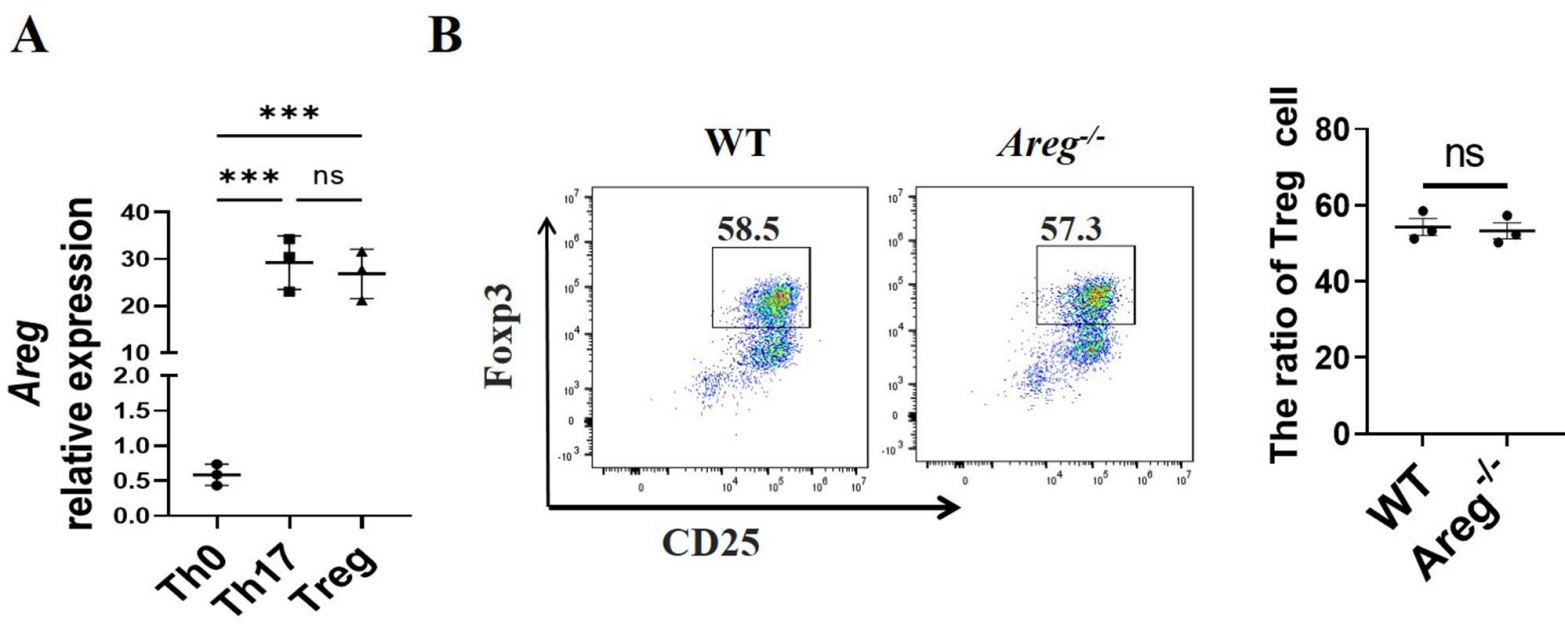
Treg cells produce high levels of AREG. (**A**) Spleen CD4^+^ T cells were isolated and cultured under neutral (Th0), Th17 and Treg-polarization conditions for 5 days, and *Areg* expression was determined by qRT-PCR. (**B**) Spleen WT and *Areg*^*−/−*^ CD4^+^ T cells were isolated and cultured under Treg conditions, Tregs ratio was detected by flow cytometry. (**A**) one-way ANOVA; (**B**) Unpaired Student’s t-test; ^***^P < 0.001; ns: not significant

Our previous studies found that Th17 cells produce high levels of AREG and Th17 cell-derived AREG is involved in intestinal fibrosis [12]. To further verify whether Treg produce AREG and its role in intestinal fibrosis, we determined *Areg* expressions in T cells, including Th0, Th17, and Tregs. CD4^+^ T cells were cultured under naïve (Th0), Th17 and Treg-polarization conditions, and the expression of *Areg* was measured using qRT-PCR. In line with our earlier research, Th0 cells showed minimal *Areg* expression, while Tregs displayed *Areg* levels comparable to those of Th17 cells (Fig. 2A). We further investigated whether AREG could regulate Tregs differentiation. We isolated and cultured WT and *Areg*^*−/−*^ CD4^+^T cells under Treg-polarization conditions and found no significant difference in the number of FOXP3^+^ cells between the two groups (Fig. 2B). The above results indicated that Tregs produce high levels of AREG, which does not affect Treg differentiation.

### AREG deficiency attenuates intestinal fibrosis

**Fig. 3:**
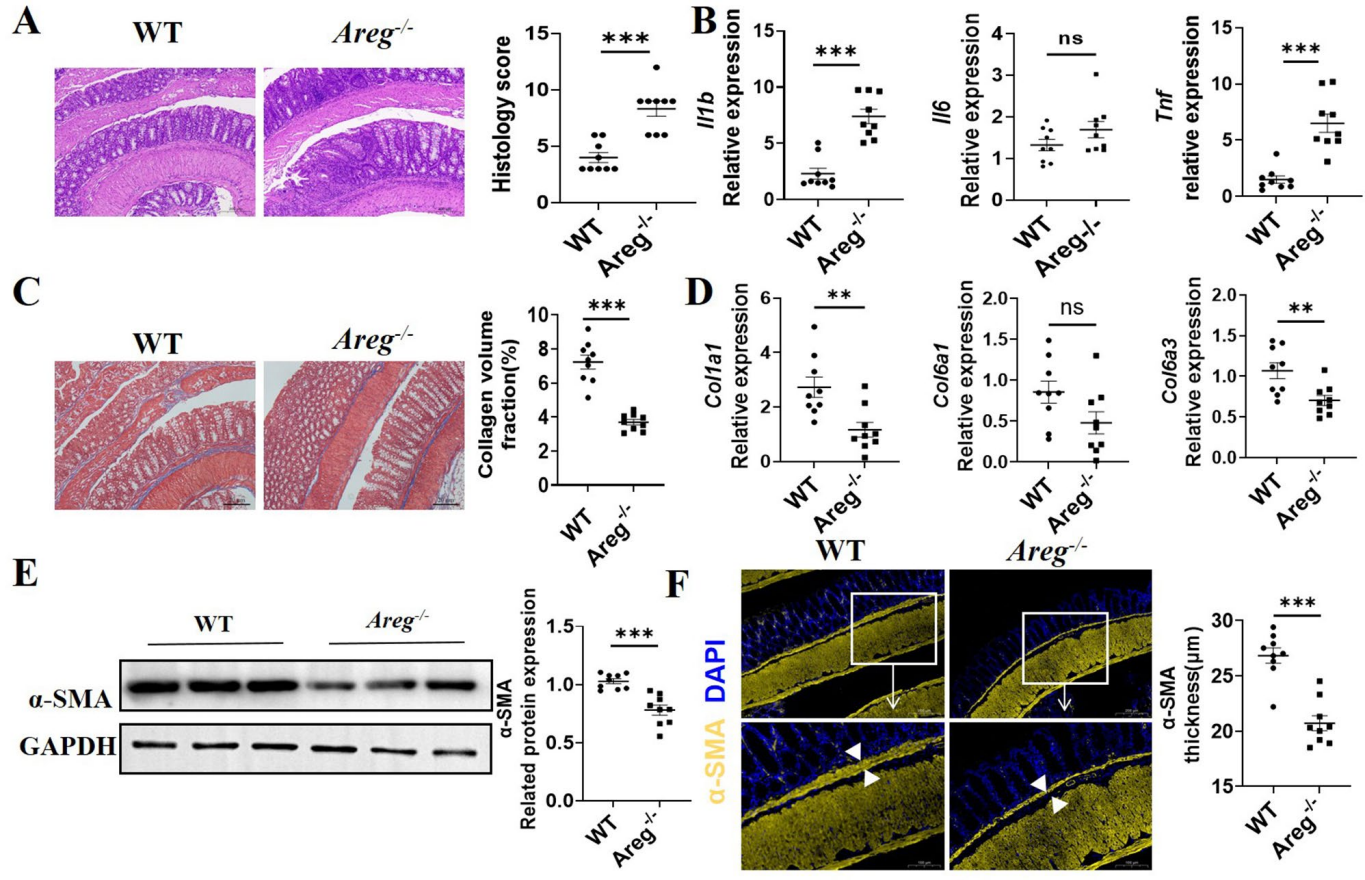
AREG deficiency attenuates intestinal fibrosis. (A-F) WT and *Areg*^*−/−*^ mice were treated with 1.5% DSS in the drinking water for 7 days and then with drinking water alone, and the above process was repeated three times. Mice were killed after a total of 3 cycles of DSS treatment (*n* = 9/group). (**A**) Disease severity was measured by histopathology and pathological scores. Scale bars, 200 μm. (**B**) *Il1b*, *Il6*, and *Tnf* levels in colonic tissues were measured by qRT-PCR. (**C**) Colon tissues were stained with Masson’s trichrome. (**D**) *Col1a1*,* Col6a1* and *Col6a3* levels in colonic tissues were measured by qRT-PCR. (**E**) α-SMA expression in colon tissues from WT and *Areg*^*−/−*^ mice was determined by western blot. (**F**) Colon tissues were stained with immunofluorescence. α-SMA layer thickness were analyzed. Representative data from 2 independent experiments with similar results. (**A**-**F**) Unpaired Student’s t-test. ^**^P < 0.01; ^***^P < 0.001; ns: not significant

To determine the role of AREG in intestinal fibrosis, we established a DSS-induced chronic colitis model to induce intestinal fibrosis in both *Areg*^*−/−*^ and WT mice. WT and *Areg*^*−/−*^ mice received 1.5% DSS in their drinking water for one week, then switched to regular drinking water, with this cycle being repeated thrice. Mice were euthanized following three rounds of DSS treatment. We observed that *Areg*^*−/−*^ mice exhibited more severe colitis than WT mice, as evidenced by higher intestinal inflammation score (Fig. 3A) and elevated expression levels of inflammatory mediators like IL-1β and TNF-α (Fig. 3B). However, *Areg*^*−/−*^ mice exhibited less severe intestinal fibrosis, as indicated by decreased collagen expression in the colon, detected via Masson’s trichrome staining (Fig. 3C). In *Areg*^*−/−*^ mice, the expression of collagen genes, such as *Col1a1 and Col6a3*, was significantly decreased (Fig. 3D). Additionally, colonic α-smooth muscle actin (α-SMA) expression and α-SMA layer thickness were also decreased in *Areg*^*−/−*^ mice (Fig. 3E-F). These findings are particularly significant as α-SMA, a specific isoform of smooth muscle actin, serves as a well-established hallmark of mature and activated myofibroblasts [19]. The reduced α-SMA expression in *Areg*^*−/−*^ mice suggests impaired myofibroblast differentiation and function, which may explain the observed attenuation of intestinal fibrosis. This is consistent with α-SMA’s crucial role in tissue contraction and ECM remodeling during the fibroblast-to-myofibroblast transition, a key process in fibrogenesis [20]. These data suggest that AREG promotes intestinal fibrosis.

### *Areg*^*−/−*^ Treg cells induce less severe intestinal fibrosis

**Fig. 4:**
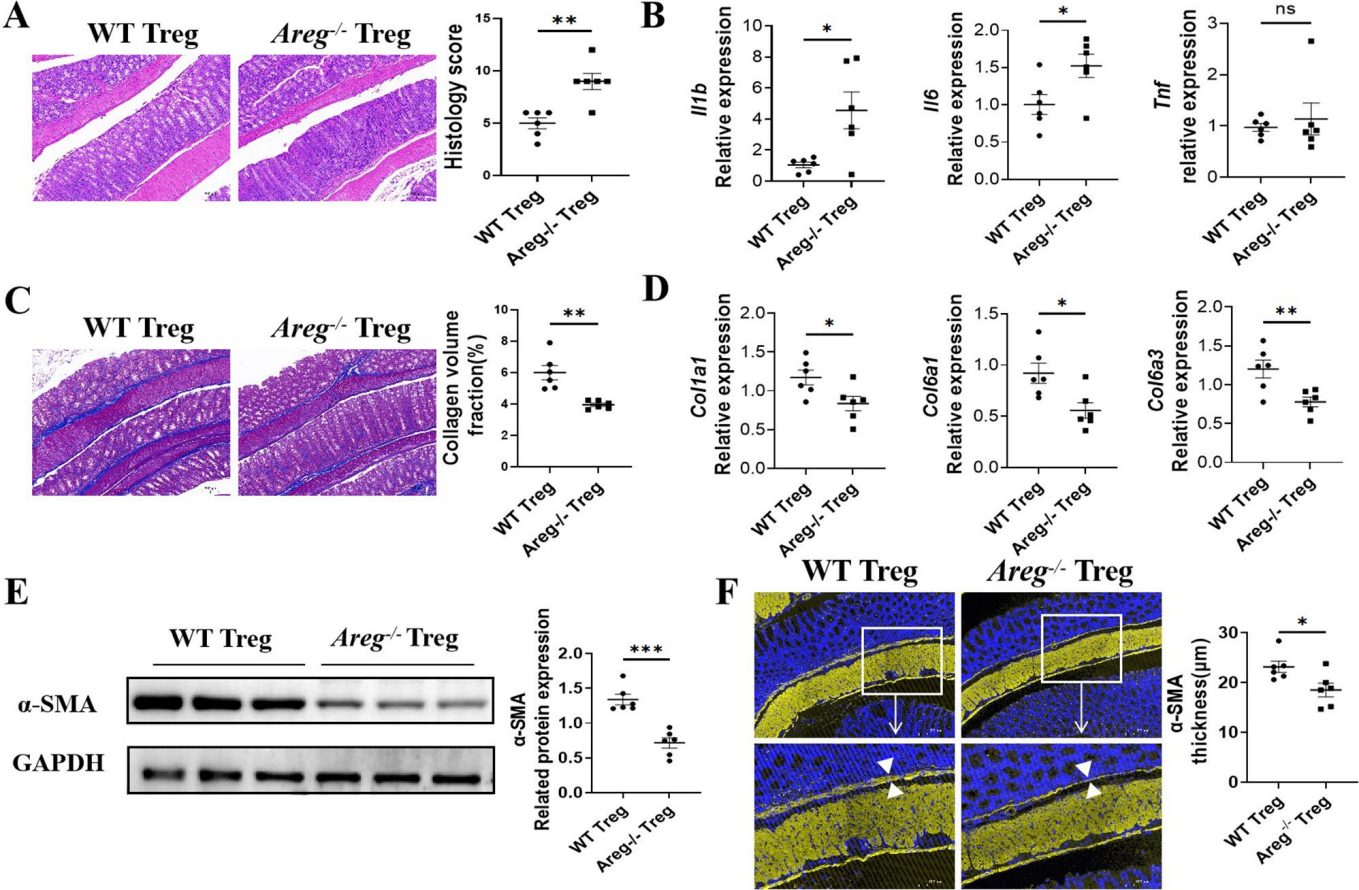
*Areg*^*−/−*^ Treg cells induce less severe intestinal fibrosis. (**A**-**F**) CD45RB^hi^ CD4^+^ T cell adoptive transfer model were used to induce chronic colitis in *Rag*^*−/−*^ mice. Meanwhile, splenic CD4^+^ T cells were isolated from WT and *Areg*^*−/−*^ mice and cultured under Treg condition for 5 days. On day 5, CD45RB^hi^ CD4^+^ T with WT or *Areg*^*−/−*^ Tregs were transferred into *Rag*^*−/−*^ mice. The mice were killed 6 weeks after cell transfer, and the severity of intestinal inflammation and fibrosis were determined (*n* = 6/group). (**A**) Disease severity was measured by histopathology and pathological scores. Scale bars, 200 μm. (**B**) *Il1b*, *Il6*, and *Tnf* levels in in colonic tissues were measured by qRT-PCR. (**C**) Intestinal fibrosis was determined by Masson staining. (**D**) *Col1a1*,* Col6a1* and *Col6a3* levels in colonic tissues were measured by qRT-PCR. (**E**) α-SMA expression in colon tissues from WT Treg and *Areg*^*−/−*^ Treg transfer mice was determined by western blot. (**F**) Colon tissues were stained with immunofluorescence. α-SMA layer thickness were analyzed. Representative data from 2 independent experiments with similar results. (**A**-**F**) Unpaired Student’s t-test. ^*^P < 0.05; ^**^P < 0.01; ^***^P < 0.001; ns: not significant

We further determined the role of Treg-derived AREG in intestinal fibrosis using the CD45RB^hi^ CD4^+^ T cell adoptive transfer model to induce chronic colitis in *Rag*^*−/−*^ mice. Meanwhile, we isolated splenic CD4^+^ T cells from *Areg*^*−/−*^ and WT mice and cultivated them under conditions that promote Treg differentiation for a period of five days. On day 5, CD45RB^hi^ CD4^+^ T cells, along with either WT or *Areg*^*−/−*^ Tregs, were transferred into *Rag*^*−/−*^ mice. Six weeks after cell transfer, the extent of intestinal inflammation and fibrosis was evaluated. The mice that transferred with *Areg*^*−/−*^ Tregs exhibited more severe colitis compared to those that received WT Tregs (Fig. 4A-B). Interestingly, *Areg*^*−/−*^ Tregs induced milder fibrosis compared to WT Tregs in *Rag*^*−/−*^ mice, as indicated by reduced deposition of colonic collagen (Fig. 4C), collagen expression (Fig. 4D), α-SMA expression and α-SMA layer thickness (Fig. 4E-F). These findings suggest that AREG facilitates Tregs induction of intestinal fibrosis independently of inflammation.

### TGF-β promotes AREG expression in Treg cells through activation of Smad3

**Fig. 5:**
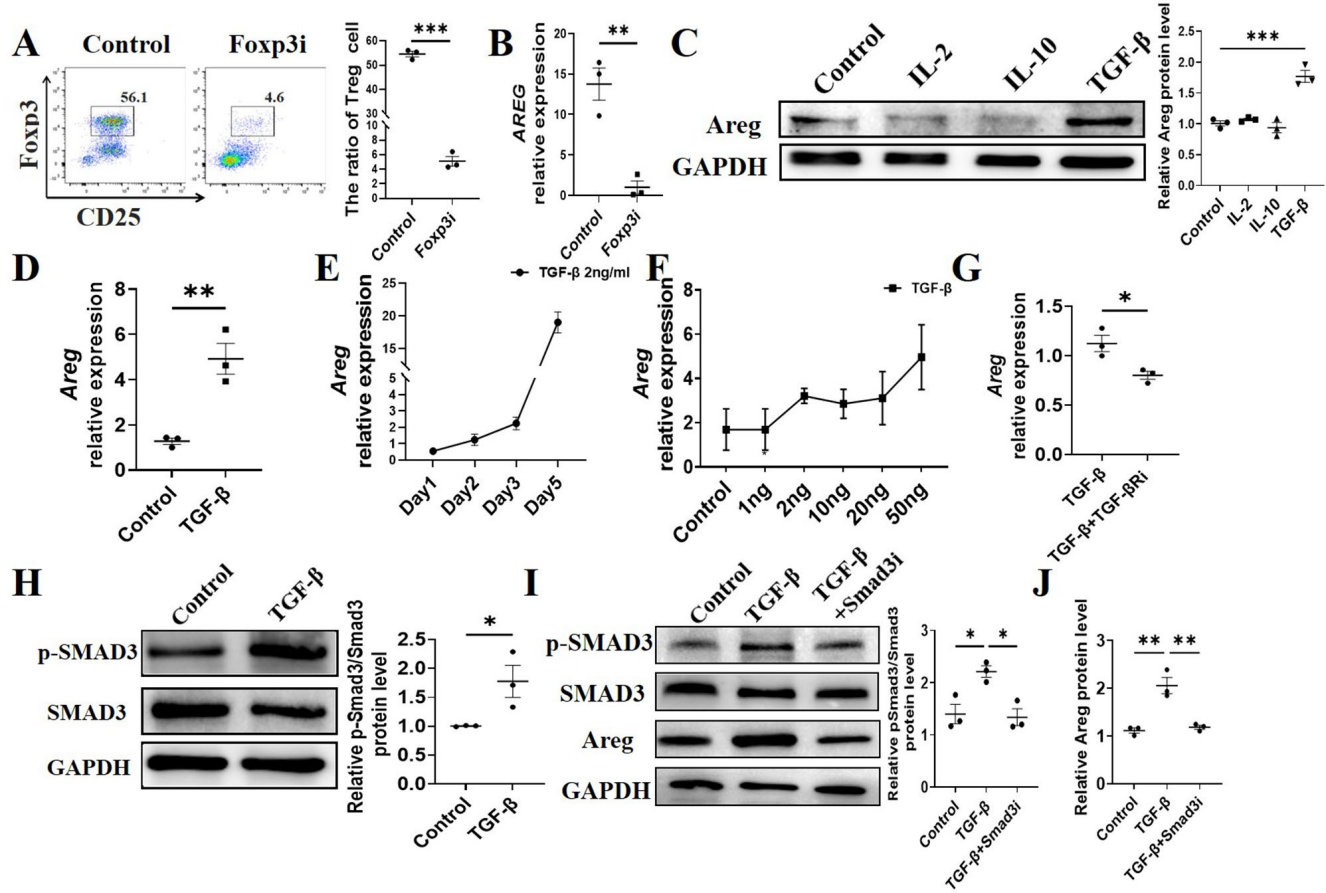
TGF-β promotes AREG expression in Treg Cells through activation of Smad3. (**A**-**B**) Spleen CD4^+^ T cells were isolated and cultured Treg-polarization conditions with or without the FOXP3 inhibitor. (**A**) Tregs ratio was determined by flow cytometry. (**B**) Areg expression was detected by qRT-PCR. (C-D) Spleen CD4^+^ T cells were isolated and cultured with or without 10 ng/ml IL-2, 10 ng/ml IL-10 or 2 ng/ml TGF-β for 5 days. (**C**) Areg expression was detected by western blot and (**D**) qRT-PCR. (**E**) Spleen CD4^+^ T cells were isolated and cultured with 2ng/ml TGF-β for 1, 2, 3, and 5 days. Areg expression was detected by qRT-PCR. (**F**) Spleen CD4^+^ T cells were isolated and cultured with TGF-β at indicated dose for 5 days. Areg expression was detected by qRT-PCR. (**G**) CD4^+^ T cells were cultured with TGF-β with or without TGF-β receptor inhibitor. Areg expression was detected by qRT-PCR. (**H**) CD4^+^ T cells were cultured with or without TGF-β for 12 h. Phosphorylated Smad3 and total Smad3 were detected by western blot. (**I**-**J**) CD4^+^ T cells were cultured with or without TGF-β and Smad3 inhibitor for 12 h. Phosphorylated Smad3, total Smad3 and AREG were detected by western blot. Representative data from 2–3 independent experiments with similar results. (**A**, **B**, **D**, **G** and **H**) Unpaired Student’s t-test; (**C**, **I** and **J**) one-way ANOVA. ^*^P < 0.05; ^**^P < 0.01; ^***^P < 0.001

We subsequently explored the mechanisms responsible for driving AREG expression in Tregs. FOXP3, a transcription factor specifically expressed in Tregs, serves as a master regulator that orchestrates Treg lineage commitment and differentiation. It is indispensable for the development of Tregs from naive CD4^+^ T cells, where it initiates and maintains the transcriptional program essential for Treg function. As a key molecular determinant, FOXP3 directly promotes Treg differentiation by regulating the expression of genes critical for Treg identity and suppressive activity [21, 22]. To assess whether the degree of Tregs differentiation affects AREG expression, we isolated splenic CD4^+^ T cells and cultured them under Treg-polarization conditions in the presence of FOXP3 inhibitor, epirubicin hydrochloride. As expected, treatment with the FOXP3 inhibitor suppressed Tregs differentiation (Fig. 5A) and also inhibited *Areg* expression in Tregs (Fig. 5B), indicating that Treg polarization enhances AREG expression in T cells.

TGF-β is a critical cytokine for Treg development [23], and the role of IL-2 in the induction of Tregs differentiation is well established [24, 25]. IL-10 is the signature cytokine of Treg [26]. To investigate whether these cytokines regulate the generation of AREG in T cells, CD4^+^ T cells were cultured in the conditions supplemented or not with TGF-β, IL-2, and IL-10 for a period of 5 days. It was observed that TGF-β, rather than IL-2 or IL-10, induced AREG protein expression (Fig. 5C). We found AREG mRNA expression level in CD4^+^ T cells was also increased after treatment with TGF-β (Fig. 5D). We further used different concentrations of TGF-β to treat CD4^+^T cells or use the same concentration of TGF-β for different times. TGF-β stimulated *Areg* expression in a manner that was both temporally and concentration-dependent (Fig. 5E-F). We added TGF-β receptor inhibitor in the Treg culture with TGF-β. The addition of TGF-β receptor inhibitor suppressed *Areg* expression (Fig. 5G).

We further explored the mechanisms through which TGF-β induce AREG expression in Tregs. Previous studies have demonstrated that TGF-β activates Smad3, which is essential for the differentiation of Tregs [25, 27]. To investigate whether TGF-β induces AREG production in Tregs via Smad3 activation, we isolated CD4^+^ T cells and cultured them with TGF-β. The results indicated a significant increase in Smad3 phosphorylation levels following TGF-β treatment (Fig. 5H). Treatment with Smad3 inhibitors resulted in reduced Smad3 phosphorylation levels (Fig. 5I) and decreased Areg expression (Fig. 5J). Collectively, these findings suggest that Smad3 is crucial for TGF-β-mediated induction of AREG in Tregs.

### Treg cell-derived AREG promotes intestinal myofibroblasts activation and proliferation

**Fig. 6:**
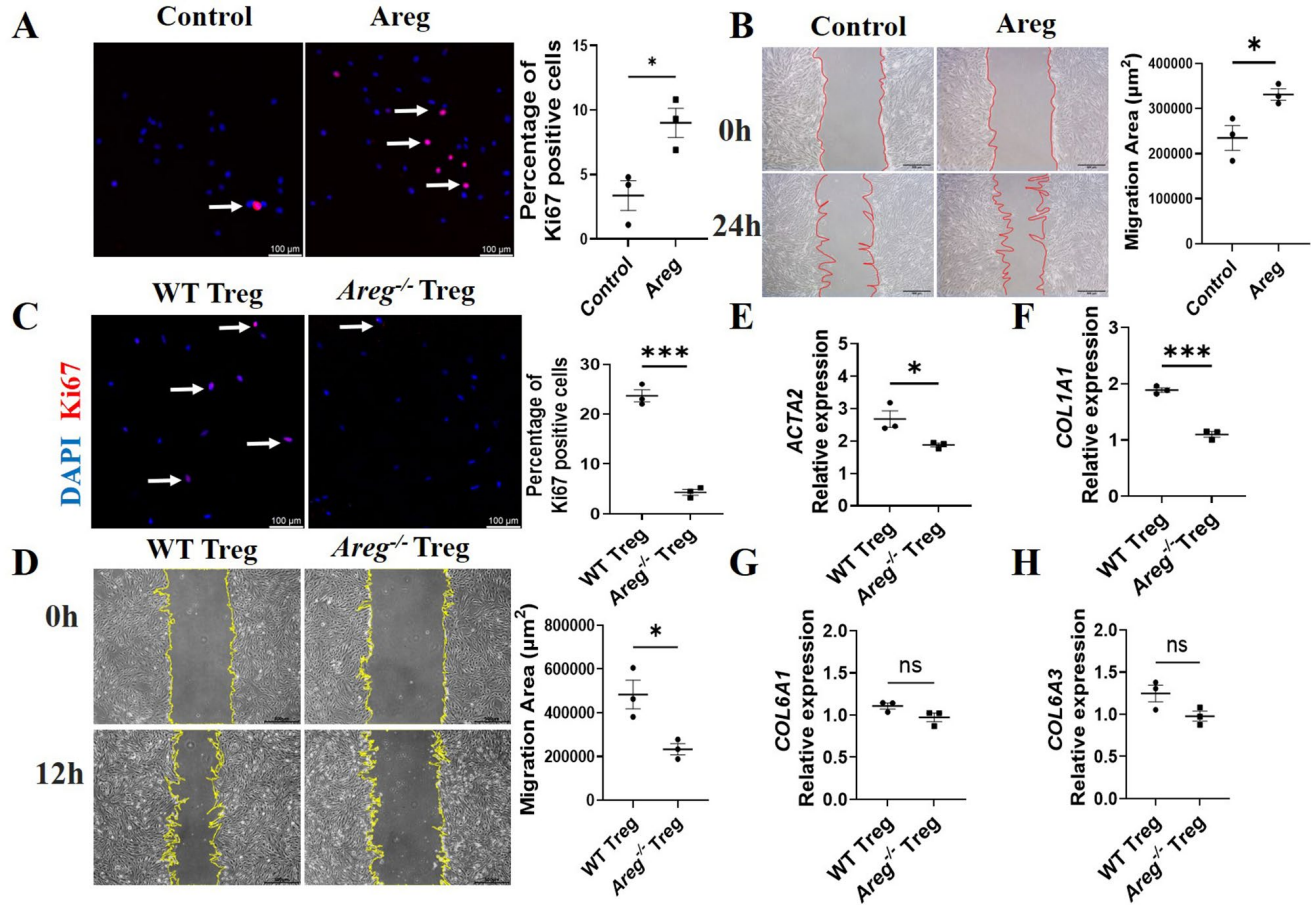
Treg cell-derived AREG promotes human intestinal myofibroblast activation and proliferation. (**A**) Human intestinal myofibroblasts (MFs) were isolated from CD patients and treated with or without 100 ng/ml AREG for 48 h, and Ki67 immunofluorescence were performed and percentage of Ki67^+^ cells were analyzed. Scale bars, 100 μm. (**B**) MFs were wounded and treated with the 100 ng/ml AREG for 24 h. Images were recorded and the extent of wound gap was analyzed by Image J. (**C**) MFs were cocultured with WT Treg or *Areg*^*−/−*^ Tregs and stained with Ki67. Percentage of Ki67^+^ cells were analyzed. (**D**) MFs were wounded and cocultured with WT Treg or *Areg*^*−/−*^ Tregs for 12 h. The extent of wound gap were analyzed by Image J. Scale bars, 500 μm. (**E**-**H**) MFs were cocultured with WT Treg or *Areg*^*−/−*^ Tregs for 3 days, *ACTA2*, *COL1A1*,* COL6A1* and *COL6A3* levels were detected by qRT-PCR. Representative data from 2–3 independent experiments with similar results. (**A**-**H**) Unpaired Student’s t-test; ^*^p < 0.05; ^***^P < 0.001; ns: not significant

The proliferation and migration of MFs play a crucial role in the development of intestinal fibrosis. We initially evaluated the pro-fibrotic effects of AREG on human intestinal MFs. The MFs were exposed to AREG and then immunostained with Ki67. Results revealed a significantly increased proportion of Ki67-positive MFs in the group exposed to AREG versus the non-treated group (Fig. 6A), suggesting that AREG promotes the proliferation of these cells. Following this, we investigated the influence of AREG on the migratory capacity of MFs by conducting a wound-healing assay. Monolayers of MFs were scratched and exposed to AREG for 24 h, after which the residual wound gap was assessed. AREG treatment resulted in a greater reduction of the wound gap compared to untreated control cells (Fig. 6B), indicating that AREG enhances MF motility.

Furthermore, we determined the role of Treg-derived AREG in the induction of the proliferation and migration of intestinal MFs. Mouse MFs were isolated and cocultured with either WT Treg or *Areg*^*−/−*^ Tregs and subsequently stained with Ki67. The proportion of Ki67-positive MFs was greater in the WT Treg treated group compared to the *Areg*^*−/−*^ Treg group (Fig. 6C). This suggests that AREG secreted by Tregs stimulates the proliferation of intestinal MFs. Additionally, we explored the influence of Treg-derived AREG on MF migration employing a wound closure assay. MF monolayers were scratched and treated with WT Treg or *Areg*^*−/−*^ Tregs for 12 h, followed by measurement of the residual wound gap. *Areg*^*−/−*^ Tregs resulted in a lesser reduction of the wound gap compared to WT Tregs (Fig. 6D), indicating that AREG enhances MF motility. α-SMA is frequently used to identify pathologic fibroblasts, with α-SMA^+^ MFs being major sources of extracellular matrix (ECM) [28]. α-SMA^−^ fibroblasts can differentiate into α-SMA^+^ fibroblasts upon stimulation by various mediators [6]. Therefore, we also examined the expression level of α-SMA mRNA (*ACTA2*) in MFs in the coculture system and found that *Areg*^*−/−*^ Tregs reduced the expression of *ACTA2* relative to WT Tregs (Fig. 6E). And *COL1A1* expression induced by *Areg*^*−/−*^ Tregs was decreased compared to WT Treg (Fig. 6F-H). These data suggest that Treg cell-derived AREG induces the proliferation, migration, and collagen expression of MFs.

### TGF-β promotes AREG expression in human intestinal myofibroblasts through activation of Smad3

**Fig. 7:**
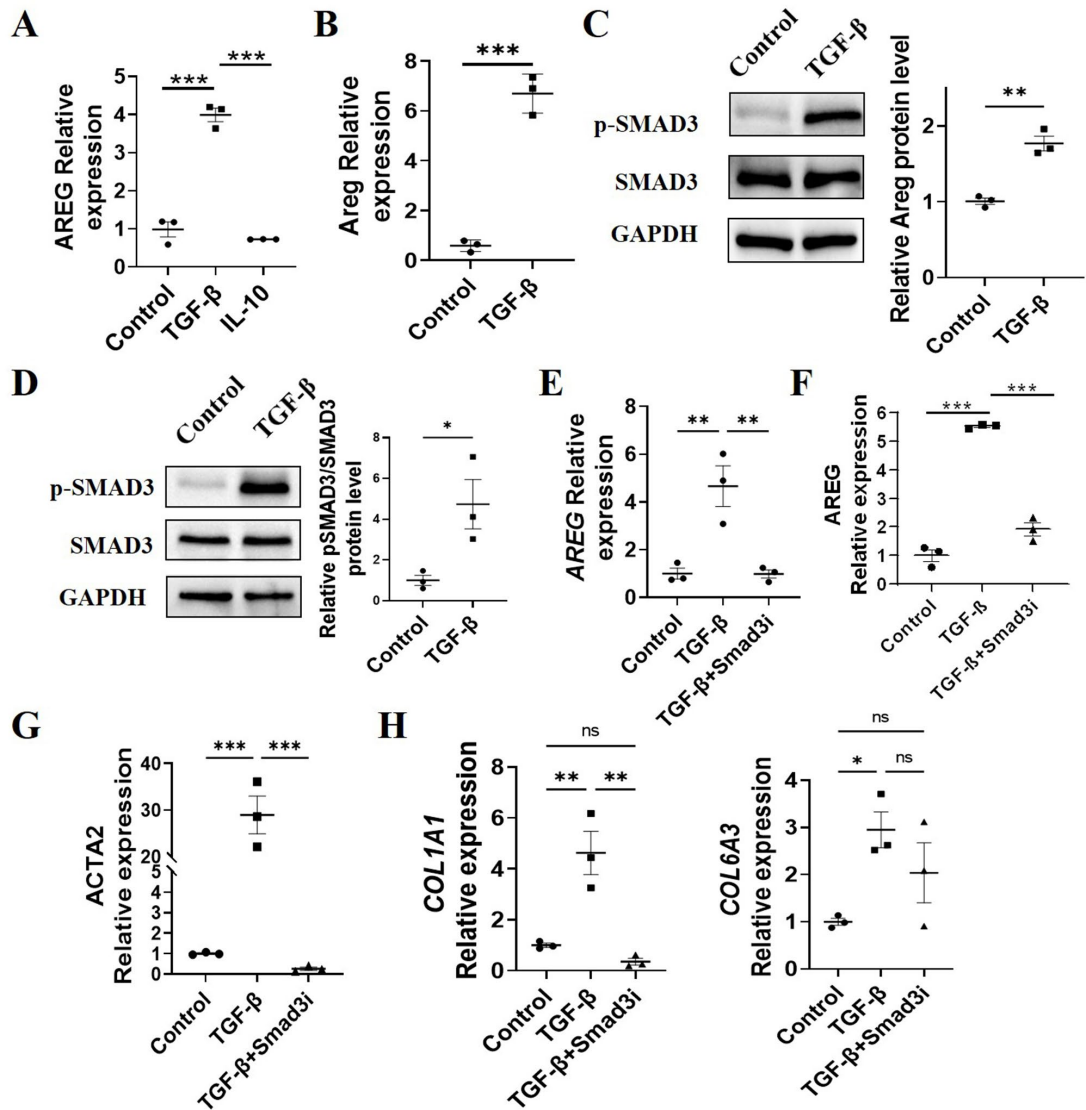
TGF-β promotes AREG expression in intestinal myofibroblasts through activation of Smad3. (**A**) Human intestinal myofibroblasts (MFs) were isolated from stenotic sites of patients with CD and treated with or without 5 ng/ml TGF-β or 10ng/ml IL-10 for 24 h. AREG expression was detected by qRT-PCR. (**B**) Human intestinal MFs were isolated from non-stenotic sites of patients with CD, treated with 5 ng/ml TGF-β. AREG expression was determined by qRT-PCR. (**C**-**D**) Intestinal MFs were isolated from stenotic sites and non-stenotic sites of patients with CD and cultured with or without TGF-β for 24 h. Phosphorylated Smad3 and total Smad3 were detected by western blot. (**E**-**F**) MFs from stenotic sites and non-stenotic sites were cultured with TGF-β with or without the Smad3 inhibitor for 24 h. AREG expression was detected by qRT-PCR. (**G**-**H**) MFs from stenotic sites were cultured with TGF-β with or without the Smad3 inhibitor for 24 h. *COL1A1* and *COL6A3* levels were detected by qRT-PCR. Representative data from 2–3 independent experiments with similar results. (**B**-**D**) Unpaired Student’s t-test; (**A**, **E**-**H**) one-way ANOVA. ^*^P < 0.05; ^**^P < 0.01; ^***^P < 0.001; ns: not significant

TGF-β induces Tregs to secrete AREG, and Treg cell-derived AREG subsequently promotes the proliferation and migration of MFs. To investigate whether TGF-β directly affects human intestinal MFs and promotes AREG expression, thereby contributing to intestinal fibrosis, we isolated human intestinal MFs from stenotic and non-stenotic sites in CD and treated them with TGF-β to assess AREG expression. AREG expression in TGF-β-treated MFs from both stenotic (Fig. 7A) and non-stenotic sites (Fig. 7B) was elevated compared to the control group. Additionally, considering IL-10 as the signature cytokine of Treg, we evaluated its role in regulating AREG production in human intestinal MFs. These MFs were cultured in the presence or absence of IL-10, and the results indicated that IL-10 did not induce AREG expression (Fig. 7A), suggesting that TGF-β specifically promotes AREG expression in MFs.

To determine whether TGF-β induces AREG production in human intestinal MFs through Smad3 activation, we isolated and cultured human intestinal MFs with TGF-β. The results indicated a significant increase in Smad3 phosphorylation levels in MFs from both stenotic (Fig. 7C) and non-stenotic sites (Fig. 7D) of CD following TGF-β treatment. Upon application of a Smad3 inhibitor, *AREG* expression was reduced in MFs from both stenotic (Fig. 7E) and non-stenotic sites (Fig. 7F). Additionally, *ACTA2* expression levels in MFs from stenotic decreased after Smad3 inhibitor treatment (Fig. 7G), and *COL1A1* expression was similarly reduced (Fig. 7H). Collectively, these data suggest that TGF-β directly influences AREG expression in MFs through Smad3 activation.

### AREG and TGF-β were increased in fibrotic sites from patients with CD

To determine the contribution of AREG from Tregs to intestinal fibrosis in CD patients, we initially gathered peripheral blood samples from individuals with CD, encompassing those with and without intestinal fibrosis, and then examined the ratio of Areg^+^ Tregs. The results indicated that the ratio of Areg^+^ Tregs in peripheral blood was higher in CD patients exhibiting intestinal fibrosis than in those without fibrosis (Supplementary Fig. 1A). Furthermore, we measured the expression levels of AREG and TGF-β in intestinal mucosal tissues from both nonfibrotic and fibrotic areas of the same CD patients. Fibrotic sites exhibited higher levels of *AREG* and TGF-β expression than nonfibrotic sites (Supplementary Fig. 1B-C). Additionally, AREG expression in fibrotic sites was positively correlated with TGF-β expression (Supplementary Fig. 1D).

## Discussion

Tregs have been implicated in the pathogenesis of intestinal fibrosis during intestinal inflammation, primarily due to their production of TGF-β [29, 30]. In this study, we demonstrated that various cell types exhibited differential expression between stricture and non-stricture CD. Notably, among T cells, Tregs constituted a larger proportion and were significantly elevated in the stenotic tissues of stricture CD. TGF-β induced both Tregs and human intestinal MFs to secrete AREG, which subsequently enhanced MF proliferation and motility. Importantly, *Areg*^*−/−*^ Tregs induced less severe fibrosis when transferred into *Rag*^−/−^ mice.

Emerging single-cell analytics broaden the avenues of investigation in CD [8, 31, 32]. Florian Rieder and colleagues performed the first single-cell RNA sequencing (scRNAseq) analysis on stenotic intestinal tissues in CD [8]. Utilizing data provided by Dr. Rieder, we reexamined the proportion of T cells in stenotic and non-stenotic regions and observed differential expression of several cell types between stricture and non-stricture CD. Among T cells, Tregs represented a larger proportion and were significantly elevated in the stenotic tissues of stricture CD. Tregs, a subset of T cells, secrete IL-10 and TGF-β, which have been implicated as anti-inflammatory agents in CD. While their profibrotic role in pulmonary fibrosis is well established, their involvement in intestinal fibrogenesis remains less defined. It has been reported that Tregs depletion in lung fibrosis attenuates fibrosis, potentially relying on the indirect effects of IL-10 and TGF-β secreted by Tregs [33]. Administration of a TGFβ1-expressing plasmid increased Tregs producing TGFβ1 and IL-10, ameliorating pulmonary fibrosis in mice [34]. However, several studies have suggested that Tregs may induce rather than suppress intestinal fibrosis [13, 35, 36]. The TGFβ1 receptor (ALK5) mediates collagen synthesis in experimental intestinal fibrosis [37]. In this study, we found that *Areg*^*−/−*^ mice developed more severe colitis than WT mice, yet exhibited less severe intestinal fibrosis.

We found that Tregs had elevated levels of AREG expression. Interestingly, *Areg*^*−/−*^ Tregs induced less severe fibrosis compared to WT Tregs. In our study, we cocultured MFs with either WT Treg or *Areg*^*−/−*^ Tregs and found that *Areg*^*−/−*^ Treg group exhibited a lower percentage of MF proliferation and migration. This indicates that Treg cell-derived AREG facilitates the proliferation, migration, and collagen expression of MFs. These findings suggest that Treg-derived AREG plays a role in the development of intestinal fibrosis.

AREG, a member of the epidermal growth factor (EGF) family [38], is expressed by a range of cells, including intestinal epithelial cells [39], dendritic cells (DC) [40], keratinocytes [41], leukocytes [12], and human intestinal MFs. AREG exerts protective effects against intestinal inflammation [42, 43]. Neutrophils facilitate the synthesis of AREG in intestinal epithelial cells, which supports the maintenance of the intestinal epithelial cell (IEC) barrier integrity and homeostasis [39]. ILC2s derived AREG ameliorated intestinal inflammation [44]. It has been documented that Th17 cells express high levels of AREG and contribute to intestinal fibrosis [12]. Our findings indicate that Tregs produce AREG at levels similar to those of Th17 cells, and AREG plays a role in the fibrotic effects induced by Tregs in the intestine. Furthermore, scRNAseq results revealed a significant increase in Tregs within the stenotic intestinal tissues of CD. Critically, peripheral blood analysis indicated elevated AREG expression in Tregs from CD patients with intestinal fibrosis, underscoring the pivotal role of Treg-derived AREG in the development of intestinal fibrosis in patients with CD.

In our study, we demonstrated that Treg polarization enhances AREG expression, which can be inhibited by a FOXP3 inhibitor. Among the cytokines that promote Tregs differentiation, TGF-β, but not IL-2, promoted Tregs production of AREG through a mechanism mediated by Smad3. IL-10, a signature cytokine of Tregs, did not affect the production of AREG in Tregs. Interestingly, TGF-β, an important cytokine for Tregs differentiation and autocrine secretion, has been reported to stimulate AREG production in intestinal epithelial cells (IECs) [39]. However, the direct effect of TGF-β on MFs remains unclear. To address this, we treated MFs with TGF-β and found that it could promote AREG production in MFs via Smad3. Our findings indicate that TGF-β can induce AREG production in both Tregs and MFs.

## Conclusions

In summary, our study showed that AREG production by Tregs mediates the development of intestinal fibrosis and regulates it through the TGF-β-Smad3-AREG axis, which is crucial in the pathology of fibrosis. Thus, our research offers a new targeted therapeutic strategy for intestinal fibrosis associated with CD.

## Electronic supplementary material

Below is the link to the electronic supplementary material.

Supplementary Material 1

## Data Availability

The study’s key findings are fully described in the main text and the accompanying Supplementary Material. For any additional questions or clarifications, please reach out to the designated corresponding authors.
