# Supplementary material for: Treg and intestinal myofibroblasts-derived Amphiregulin induced by TGF-β mediates intestinal fibrosis in Crohn’s disease
Source: J Transl Med. 2025 Apr 17;23:452. doi: 10.1186/s12967-025-06413-6 (PMC12004752; doi:10.1186/s12967-025-06413-6)
Supplement: Supplementary file 1 — Supplementary Material 1 [file 12967_2025_6413_MOESM1_ESM.docx]

**Treg and Intestinal Myofibroblasts-Derived Amphiregulin Induced by TGF-β Mediates Intestinal Fibrosis in Crohn’s Disease**

Lu, Wang, et. al.

**Supplemental Methods**

**Extraction of primary human intestinal fibroblasts**

Intestinal specimens were collected from patients with CD undergoing surgery for intestinal strictures. Tissue samples measuring 3*3 cm from both narrow and non-narrow areas. The mucosal epithelial layer, muscularis mucosae, and serosal layer were removed, leaving the laminae propria intact. The laminae propria was cut into 2-3 cm strips and washed in dithiothreitol (DTT) solution (75 mg DTT dissolved in 50 ml HBSS) for 15 minutes. The strips of laminae propria were incubated in HBSS solution containing 2.5% antibiotics for 3 hours at room temperature, with flipping every 20 minutes.10 cm cell culture dishes were prepared and divided into 20*20 mm squares using a dissecting knife. The washed laminae propria strips were cut into 1*1 mm pieces, with each piece placed in a square and a small amount of Dulbecco's Modified Eagle Medium (DMEM) comprising 10% fetal bovine serum added. The dishes were then placed overnight in a 37°C cell culture incubator. 10ml of complete culture medium was added on the second day. Fibroblasts were observed migrating from the edges of the tissue within 4-7 days. Once fibroblasts were clearly present, tissue blocks were removed. When cells had sufficiently grown, they were digested into single-cell suspension using trypsin for cryopreservation and passage.

**Western Blot**

The total protein was extracted from intestinal tissues, fibroblasts and T cells using RIPA containing protease inhibitor cocktail. The concentration of protein was measured by bicinchoninic acid (BCA) method. Protein samples were separated by 10% SDS-PAGE and transferred to PVDF membranes. The blots were·blocked with 5% skim milk for 1 hour at room temperature after electrotransfer. And then the blots were incubated at 4°C overnight with primary antibody (Areg, 1:1000, Abmart, PU595967S; pSMAD3, 1:1000, MedChemExpress, HY-P80477; SMAD3, 1:1000, MedChemExpress, HY-P80325; α-SMA, 1:1000, Santa Cruz Biotechnology, sc-53142; GAPDH, 1:10000, Proteintech, 60004-1-Ig). After washing, the blots were further incubated with the corresponding secondary antibody (Anti-rabbit IgG-HRP, 1:10000, Bioworld, BS13278; Anti-mouse IgG-HRP, 1:10000, Bioworld, BS12478) and detected using the Tanon-4600 ChemiDoc Imaging System.

**Quantitative reverse-transcriptase polymerase chain reaction (qRT-PCR)**

The total·RNA was extracted from cells or tissues using Trizol. The RNA content and quality were determined with Nanodrop. Reverse transcription of RNA into cDNA synthesis with a Reverse Transcription kit (Vazyme Biotech, R323-01). qRT-PCR was performed with the SYBR Green qPCR Master Mix (Vazyme Biotech, Q341-02) in a Step One Plus Real-time PCR System (Applied Biosystems). Primers were designed and ordered from Tsingke Biotech and normalized against GAPDH·mRNA expression. All the primers used in this study·were listed·in Supplementary·Table·2.

**Flow cytometry**

T cells were stimulated with Cell Stimulation Cocktail (plus protein transport inhibitors) (eBioscience) for 5 hours at 37˚C. And then, anti-mouse CD4-FITC (Biolegend), anti-mouse CD25-APC (Biolegend) or anti-human CD4-FITC (Biolegend), anti-human CD25-APC (eBioscience) were used for surface staining. After washing, cells were fixed and permeabilized using the FOXP3/Transcription Factor Staining Buffer Set (eBioscience). Intracellular staining was stained with anti-mouse Foxp3 PE (eBioscience), anti-human Foxp3 PE (eBioscience) and anti-human Areg APC (eBioscience).

**Immunofluorescence staining**

The prepared paraffin sections were dewaxed and antigen retrieval was performed. The adherent cell was fixed by 4% paraformaldehyde. Then, they were sealed with goat serum after penetration. They were subsequently incubated with primary antibody (α-SMA 1:100, Santacruz, SC-53142; Ki67, 1:1000, Abcam, ab15580) overnight at 4℃, followed by Alexa Fluor 488 (or 594)- labelled secondary antibody (1:1000, Jackson, 115-545-003 or 111-585-003) for 1 h at room temperature in the dark. Then, DAPI (Biosharp, BL105A) and antifade mounting medium were added in turn. Finally, the slides were covered by coverslips. Imaging was recorded with Thunder Imager microscope (Thunder, DMi8).

**Scratch wound-healing assay**

Human intestinal myofibrobalsts were plated in a 6-well plate until it covered 90% the bottom of the plate. The monolayer of cells was scratched with a 200μl pipette tip to form a straight line, and the floating cells were removed by washing with PBS. They were co-cultured with WT Treg or *Areg^-/-^* Treg cells at a ratio of 1:10. After 3 days of co-culture, the suspended Treg cells were washed with PBS, and the gaps were photographed with an inverted microscope (OlympusCKX41).

**scRNAseq data analysis**

Data analysis refers to the article published by Belinda Phipson et al [1]. Differential abundance testing was performed for each cluster across stricture CD and nonstricture CD using propeller method (part of the speckle v1.0.0 package)，and using Benjamin and Hochberg procedure for FDR control (extremely significant difference was defined as FDR<0.01).


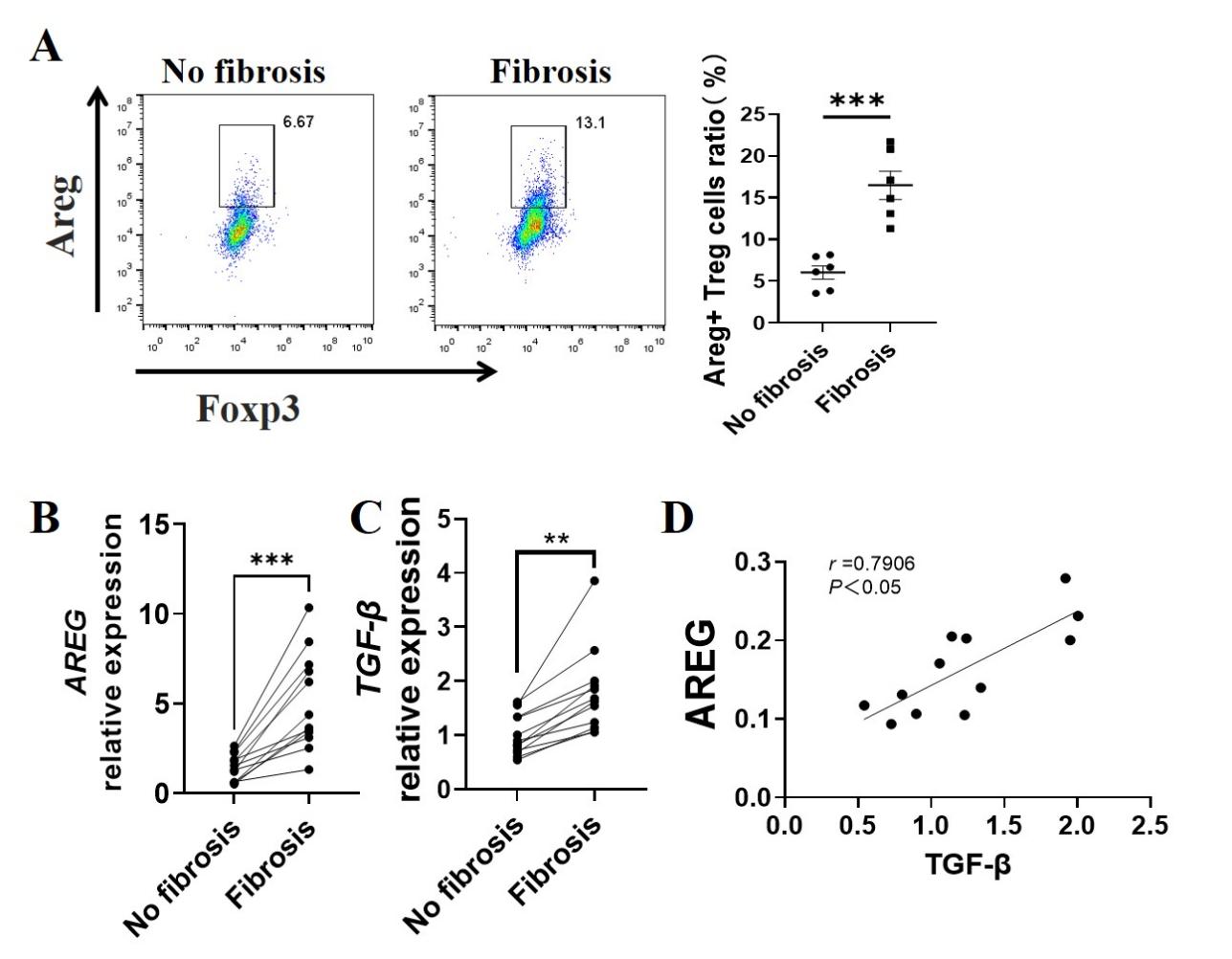


**Supplemental Figure 1. AREG and TGF-β were increased in ﬁbrotic sites from patients with CD.** (A) Peripheral blood was collected from patients with CD with or without intestinal ﬁbrosis. The ratio of Areg+ Treg cells was measured. (B-C) Nonﬁbrotic and ﬁbrotic intestinal tissues were collected from the same patients with CD. AREG and TGF-β expression in nonﬁbrotic and ﬁbrotic sites of patients with CD with intestinal ﬁbrosis was detected by qRT-PCR. (D) Correlation analysis was performed between AREG and TGF-β expression in ﬁbrotic sites. ^*^*P* < 0.05; ^**^*P* < 0.01; ^***^*P* < 0.001.

**Supplemental Table 1. The clinical characteristics of subjects.**

**
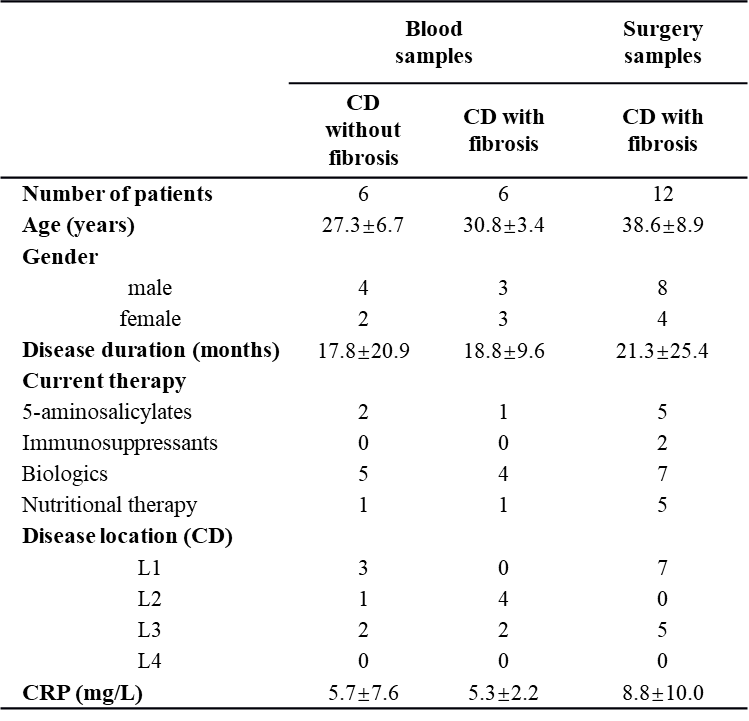
**

**Supplemental Table 2. The primers used in qRT-PCR**


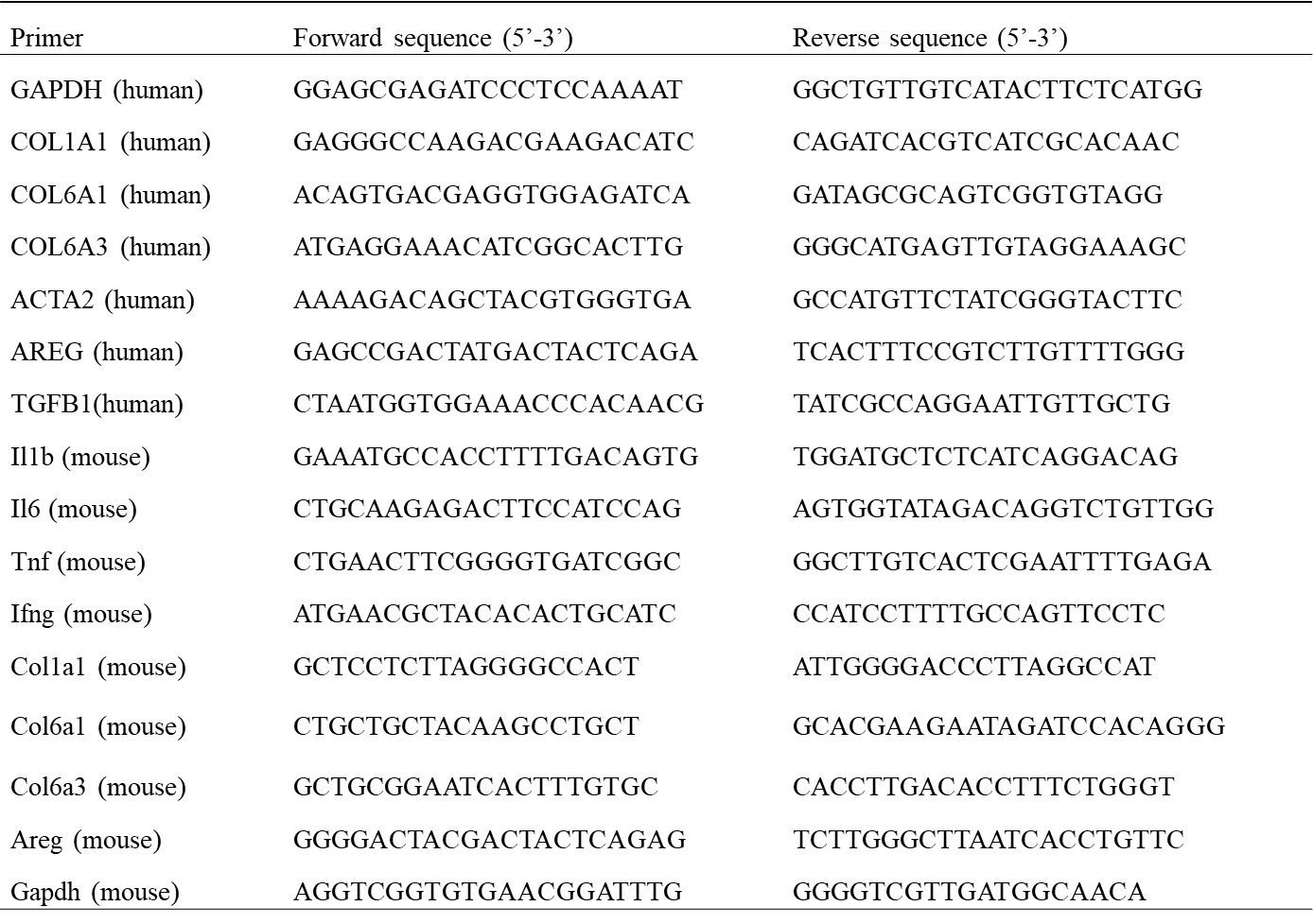


References:

1. Phipson, B., et al. propeller: testing for differences in cell type proportions in single cell data. Bioinformatics. 2022;38(20): 4720-4726.
